# Copper oxides supported sulfur-doped porous carbon material as a remarkable catalyst for reduction of aromatic nitro compounds

**DOI:** 10.1038/s41598-024-55216-0

**Published:** 2024-03-06

**Authors:** Marzie Amirjan, Firouzeh Nemati, Zeinab Elahimehr, Yalda Rangraz

**Affiliations:** Department of Chemistry, Semnan University, Semnan, 35131-19111 Iran

**Keywords:** Catalysis, Green chemistry

## Abstract

Synthesis and manufacturing of metal–organic framework derived carbon/metal oxide nanomaterials with an advisable porous structure and composition are essential as catalysts in various organic transformation processes for the preparation of environmentally friendly catalysts. In this work, we report a scalable synthesis of sulfur-doped porous carbon-containing copper oxide nanoparticles (marked Cu_x_O@CS-400) via direct pyrolysis of a mixture of metal–organic framework precursor called HKUST-1 and diphenyl disulfide for aromatic nitro compounds reduction. X-ray diffraction, surface area analysis (BET), X-ray energy diffraction (EDX) spectroscopy, thermal gravimetric analysis, elemental mapping, infrared spectroscopy (FT-IR), transmission electron microscope, and scanning electron microscope (FE-SEM) analysis were accomplished to acknowledge and investigate the effect of S and Cu_x_O as active sites in heterogeneous catalyst to perform the reduction-nitro aromatic compounds reaction in the presence of Cu_x_O@CS-400 as an effective heterogeneous catalyst. The studies showed that doping sulfur in the resulting carbon/metal oxide substrate increased the catalytic activity compared to the material without sulfur doping.

## Introduction

Heterogeneous catalysts are one of the strategic designs of chemical research in the industrial manufacturing of many fine chemicals due to their recyclability^1–7^. In recent decades, disparate species of heterogeneous catalysts have been developed and used. Among them, porous carbon/metal oxide materials have attracted considerable interest as extraordinary catalysts because of their innate structural attributes such as thermal stability, high surface area, adjustable porous structure, and beneficial mechanical properties^7–12^. However, the porous carbon-based catalysts, due to the inherent inertia of the surface chemistry of the genuine carbon materials, have suffered from special restrictions on morphology, specific surface area, and size controls, which have limited their catalytic applications^2,8,10,12–14^.

Accordingly, researchers in recent decades, in order to solve the above problems, have used heteroatoms doping into a porous carbon matrix and carbon framework that can present completely different catalytic properties^5,15–18^. Thus, specifically, non-metal heteroatoms (such as B, N, S, P, and halogen) are doped into the sp^2^ lattice of graphitic carbon. As a result, the distinction in electronegativity between carbon and heteroatoms causes them to adjust their optoelectronic and chemical properties^15,17,19–21^. Hence, heteroatoms, depending on their chemical configuration in the carbon matrix, can increase the interaction between the metal species and the support and lead to the production of new catalytic active sites, which is beneficial for the enhancement of catalytic reactivity in organic reactions^5,14,22–25^. Among different heteroatoms, the doping of sulfur is similar to that of nitrogen, and in some cases, it has a better catalytic conclusion. In comparison to nitrogen, due to the larger covalent radius of sulfur, the presence of a sulfur atom (atomic size = 102 pm) inside the carbon lattice can significantly raise the interlayer space in the carbon network. So, enlarging the interlayer distance in carbon network by doping heteroatoms with large-radius is an effective approach that significantly increases the basic sites, promoting the adsorption of organic compounds^15–17,26,27^.

Thus, it is critical to use a sensible design and the right synthesis method to produce heteroatom-doped porous carbon/metal oxide-based catalysts with two essential characteristics: a regular porous structure and a large specific surface area^9,27–33^. Since they combine metal ions (or metal categories) with organic linkers, metal–organic frameworks (MOFs), also known as porous coordination and polymers (PCPs), have garnered a lot of attention. These features include an ordered porous structure, a large specific surface area, high-grade electrical conductance, adjustable porosity, an adjustable numerical structure, and superior resistance to heat and chemicals^30,31,34,35^. Therefore, MOFs have emerged as a category of promising precursors for synthesizing carbon-based materials via different temperatures of pyrolysis^17,25,31,36^. This synthesis strategy of carbon substrates derived from MOFs can not only increase metal loading and thus the number of catalytic active sites such as Cu_x_O, but also furnish a homogenous dispensation of heteroatoms, metals, and metal oxides inside the pores of carbon-based materials^15,17,20,37,38^. As a result, porous carbon/metal oxide materials derived from MOFs effectively maintain the pore construction and large specific surface area of MOF precursors and generally have lower densities, more exposed active sites like Cu_x_O, and higher stability, thereby maximizing their performance^10,14,15,38–42^. 

for the first time, copper oxides/sulfur doped carbon-based catalyst (denoted as Cu_x_O@CS-400) have been synthesized with different ratios of diphenyl disulfide as sulfur and carbon source, using HKUST-1 as a precursor under an inert atmosphere and moderate-temperature pyrolysis conditions, and the nitro aromatic reduction was used to gauge its effectiveness. We have devised a successful method for converting unstable HKUST-1 into stable organic framework materials using the decarboxylation reaction under mild annealing conditions. During this process, the fragile coordination bonds in HKUST-1 were replaced with stable covalent C–C bonds, which show better catalytic properties from the parent MOF. Furthermore, in situ sulfur doping improves the material’s properties.

**Figure 1:**
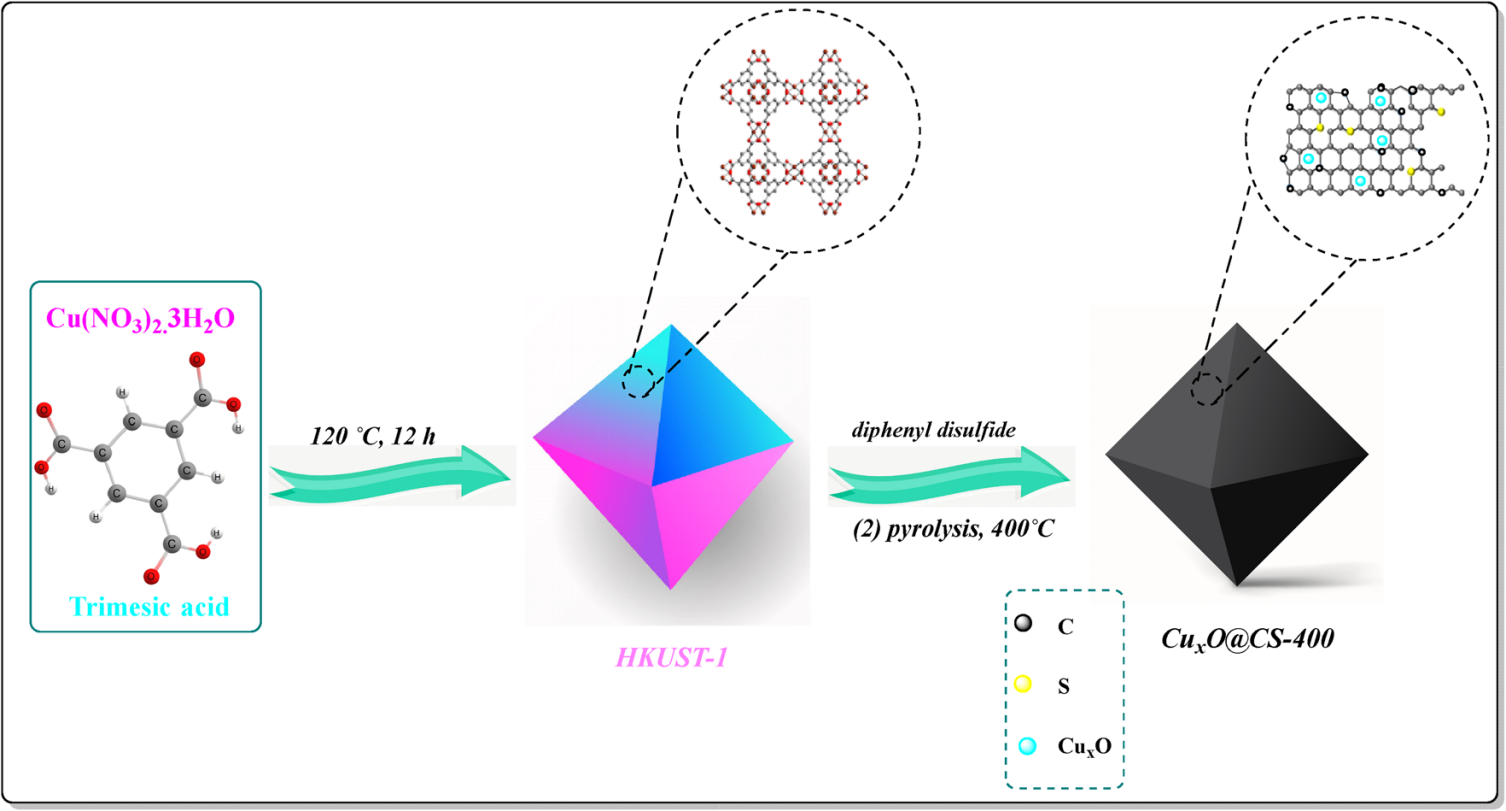
Schematic Pathway of synthesis of Cu_x_O@CS-400.

In continuation of our antecedent researches and studies in the field of synthesis and design of the novel heterogeneous catalysts^1,2,43,44^, and also their application in various synthesis reactions including the reduction of nitroaromatic compounds^1,2,44–46^, in this article, we have synthesized sulfur-doped porous carbon/copper oxides -based catalyst (denoted as Cu_x_O@CS-400) with different ratios of sulfur using HKUST-1 as a precursor under an inert atmosphere at moderate-temperature pyrolysis conditions, which Cu_x_O@CS-400 catalyst demonstrated desirable catalytic activity in the reaction of nitroaromatic compounds and its derivatives with NaBH_4_ as a reducing factor in very short reaction times and high yields. The general synthesis cycle of the catalyst is illustrated in Figure 1, and details of the laboratory process are described in the experimental department. Briefly, at first, HKUST-1 was synthesized according to the articles. HKUST-1 is a consonant polymer composed of copper ions and 1,3,5-benzentricarboxylate (BTC) ligands, with a special character area of more than 1021.2 m^2^/g. Eventually, Cu_x_O@CS-400 was drafted by moderate-warmth pyrolysis of the admixture of sulfur-doped porous carbons (which was outsourced from HKUST-1) and diphenyl disulfide.

## Experimental section

### Chemicals

All chemicals used in the synthesis of Cu_x_O@CS-400 and reduction reactions were purchased from Sigma-Aldrich. And reagents and solvents were used directly without renewed refinement.

### Devices

Identifying organic and inorganic materials was accomplished by the Furrier Transform Infrared Spectroscopy (FT-IR, 8400S). The X-ray diffraction (XRD, Bruker D8-ADVANCE diffractometer: Cu/Kα (λ = 0.15406 nm)) was utilized to analyze the crystal structure of solid specimens. The elemental combination and homogeneous dispensation of instances were distinguished via energy-dispersive X-ray (EDX) and elemental mapping procedures. The morphology and structural attributes of the specimens were appraised per the passing electron microscope or magnifier (TEM, Philips EM208S 100 kV) and scanning electron microscope (FE-SEM, TESCAN MIRA3). The Braeuer–Emmett–Teller (BET) analysis for the measurement of the specific area and porosity distribution of the solid sample was provided at a low temperature (150 °C) via N_2_ adsorption–desorption isotherms (Belsorp Mini II).

### The procedure for the synthesis of, porous carbon/copper oxides catalyst derived from MOFs

#### Synthesis of HKUST-1

HKUST-1 was synthesized from copper (II) nitrate trihydrate (Cu(NO_3_)_2_ 3H_2_O) and 1,3,5-benzenetricarboxylic acid (C_9_H_6_O_6_) based upon the reported methodology^47^. Specifically, Cu (NO_3_)_2_ 3H_2_O (2.174 g, 8.998 mmol) was dissolved in 30 mL distilled water; in the meantime, benzene-1,3,5-tricarboxylic acid (1.05 g, 4996 mmol) as an organic ligand was dissolved in 30 mL ethanol. Subsequently, the solutions were blended together and transferred to a 100 mL round bottom flask, and the mixture was continuously agitated for 30 min under an intense stirring situation at ambient temperature. After stirring for 30 min, the mixture was transferred to a 100 mL Teflon-lined stainless-steel autoclave and kept at 120 °C for 12 h. Eventually, after cooling to room temperature, the blue powder was collected by centrifugation, and washed several times with deionized water and ethanol alternately, and dried at 60 °C in a vacuum for 12 h, to modify the synthesized HKUST-1, the obtained blue powder was chemically purified with ethanol and dichloromethane, and finally, in order to remove solvent molecules and activate the synthesized HKUST-1, the solid powder was heated in an oven at 150 °C for 12 h.

#### Synthesis of Cu_x_O@CS-400

The activated MOF powder (HKUST-1) and Diphenyl disulfide at different ratios (H:D 1:2, 1:3, and 1:5) were dissolved in 15 mL of *N*,*N*-dimethylformamide. The mixture was stirred for 24 h and dried in an oven at 60 °C. The precursor prepared was carbonized straight under the nitrogen atmosphere at 400 °C for 2 h with a thermal rapidity of 10 °C min^−1^ under the N_2_ atmosphere. Finally, the above-carbonized sample was labeled Cu_x_O@CS-400 and was used as a catalyst with excellent efficiency in organic reactions, and as expected, the catalyst has significant recyclability and can run eight times without any obvious decline in catalytic activity. Figure 1 epitomizes the schematic representation of the synthesis methodology of the carbon-based copper oxide catalyst. The Cu_x_O@C-400 was synthesized accordingly, without diphenyl disulfide.

#### Catalytic test

1mmol of substrate, 3 mL of deionized water and 7 mg of Cu_x_O@CS-400 were stirred for a few minutes at ambient temperature. Following, NaBH_4_ (2 mmol) as the reductant was added to the above mixture, and the reaction mixture was stirred at 55 °C. The progression of the hydrogenation reaction was followed by TLC. At the end of the reaction time, the Cu_x_O@CS-400 was separated, rinsed, and reused. The catalyst was isolated, the resultant catalyst-free mixture was extracted by EtOAc as an organic phase (3 × 5 mL). The organic (EtOAc) layer was separated and dried by sodium sulphate. Eventually, by vaporizing the solvent, the pure product was gained (Table 1).

**Table 1:** Hydrogenation of diverse nitroarenes on Cu_x_O@CS-400 subject optimum conditions^. a, b^


Entry	product	Time (min)	Yield (%)	TOF^c^ (mol g^−1^ h^−1^)	TON^d^
1		35	99	3763	131705
2		45	99	3712	167,040
3		35	99	6951	243,285
4		60	99	4609	276,540
5		30	98	6355	190,650
6		20	100	7724	154,480
7		50	98	3784	189,200
8		25	98	7007	175,175
9		35	96	3411	119,385
10		40	99	7099	283,960

^a^Reaction condition: substrate (1 mmol), NaBH_4_ (2 mmol), H_2_O (3 mL), Cu_x_O@CS-400 (7 mg).

^b^Isolated yield.

^c^TOF $$=\frac{ mol of converted substrate }{g of catalyst*reaction time (h)}$$×100.

^d^TON$$=\frac{ yield of product }{mol copper in catalyst}$$.

#### Some spectra data

Aniline (Table 1, entry 1), light yellow liquid, ^1^H NMR (300 MHz, CD_3_Cl) δ: 7.12 (t, *J* = 7.8 Hz, 2H), 6.70(t, *J* = 7.2 Hz, 1H), 6.62(d, *J* = 7.5 Hz, 2H), 3.41 (br s, 2H).

4-Aminobenzonitrile (Table 1, entry 2) White powder, M.P. 85 °C, ^1^H NMR (300 MHz, CD_3_Cl) δ: 7.39(d, *J* = 7.8 Hz, 2H), 6.65(d, *J* = 7.5 Hz, 2H), 3.5 (br s, 2H).

4-Bromo aniline (Table 1, entry 3) White powder, M.P. 65–66 °C, ^1^H NMR (300 MHz, CD_3_Cl) δ:7.21(d, *J* = 8.2 Hz, 2H), 6.54(d, *J* = 8.4 Hz, 2H), 3.78 (br s, 2H).

4-Chloro-2-trifluoromethyl aniline (Table 1, entry 4), Pinkish crystal, M.P.: 35–38 °C, ^1^H NMR (300 MHz, CD_3_Cl) δ:7.39 (s, 1H), 7.23 (d, *J* = 8.5 Hz, 1H), 6.62 (d, *J* = 8.7 Hz, 1H), 3.99 (br s, 2H).

*o*-Phenylenediamine (Table 1, entry 5), Yellowish crystal, M.P.: 101–103 °C, ^1^H NMR (300 MHz, CD_3_Cl) δ:6.68(m, 4H), 3.31 (br s, 2H).

4-Aminophenol (Table 1, entry 8) Light brown powder, M.P.: 185–189 °C, ^1^H NMR (300 MHz, CD_3_Cl) δ:8.42 (br s, 1H), 6.4–6.49 (m, 4H), 4.32 (br s, 2H).

4-Aminobenzaldehyde (Table 1, entry 9) Slight yellow crystal, M.P.: 68–71 °C, ^1^H NMR (300 MHz, CD_3_Cl) δ:9.76 (s, 1H), 7.57 (d, *J* = 8.4 Hz, 2H) s, 1H), 6.65 (d, *J* = 8.1 Hz, 2H), 4.85 (br s, 2H).

## Results and discussion

### Preparation of Cu_x_O@CS-400

Figure 1 illustrates the process to prepare Cu_x_O@CS-400 catalyst, which was synthesized by thermal pyrolysis of a mixture of HKUST-1 and diphenyl disulfide at 400 °C for 2 h. As shown in Figure 1, firstly, the HKUST-1 was constructed according to the literature^47^. In the second step, the sulfur heteroatom was infiltrated into porous HKUST-1 by thermally annealing the precursor, which had been prepared by the wet-impregnation technique of a mixture of Ph_2_S_2_ and HKUST-1in three ratios. After carbonization of the pre-materials in a stationary atmosphere, further organic connections were thermally transmuted into a porous carbon matrix, and Cu_x_O@CS-400 was constructed as a heterogeneous catalyst.

### Characterization of HKUST-1 and Cu_x_O@CS-400

#### FT-IR analysis

**Figure 2:**
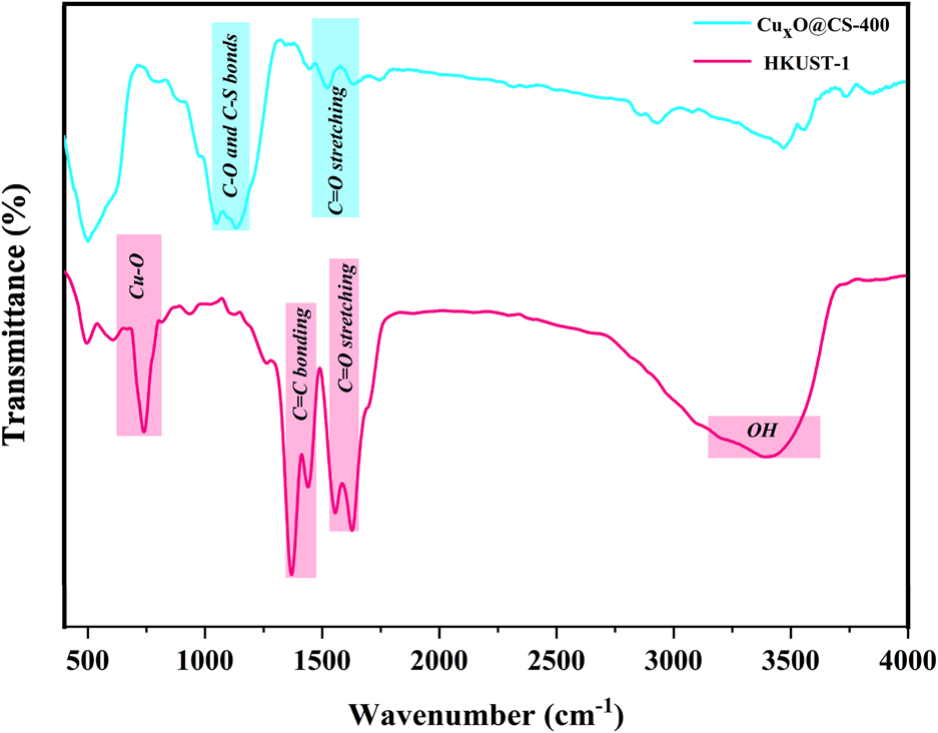
The FT-IR spectra HKUST-1 and Cu_x_O@CS-400 catalyst.

In order to explain the architecture of the Cu_x_O@CS-400–2 catalyst, the FT-IR spectrometric study was carried out. In Fig. 2, the FT-IR spectrum of HKUST-1 is presented. Peaks related to C=O asymmetric and symmetric stretching vibrations of carboxylic ligands appeared in 1646 cm^−1^ and 1375 cm^−1^. The C=C groups of the aromatic rings were observed at 1431 cm^−1^, which demonstrated that HKUST-1 has been successfully synthesized^47,48^. On the other hand, obviously, the XRD analysis confirmed the prosperous preparation of HKUST-1. After the pyrolysis of the precursor, due to the production of carbonaceous material, the intensity of C=O was decreased by a considerable amount, and some peaks appeared in the distinctness of 1100–1200 cm^−1^, which was associated with the C–O and C–S bonds in the as-synthesized Cu_x_O@CS-400 catalyst.

#### XRD measurement

**Figure 3:**
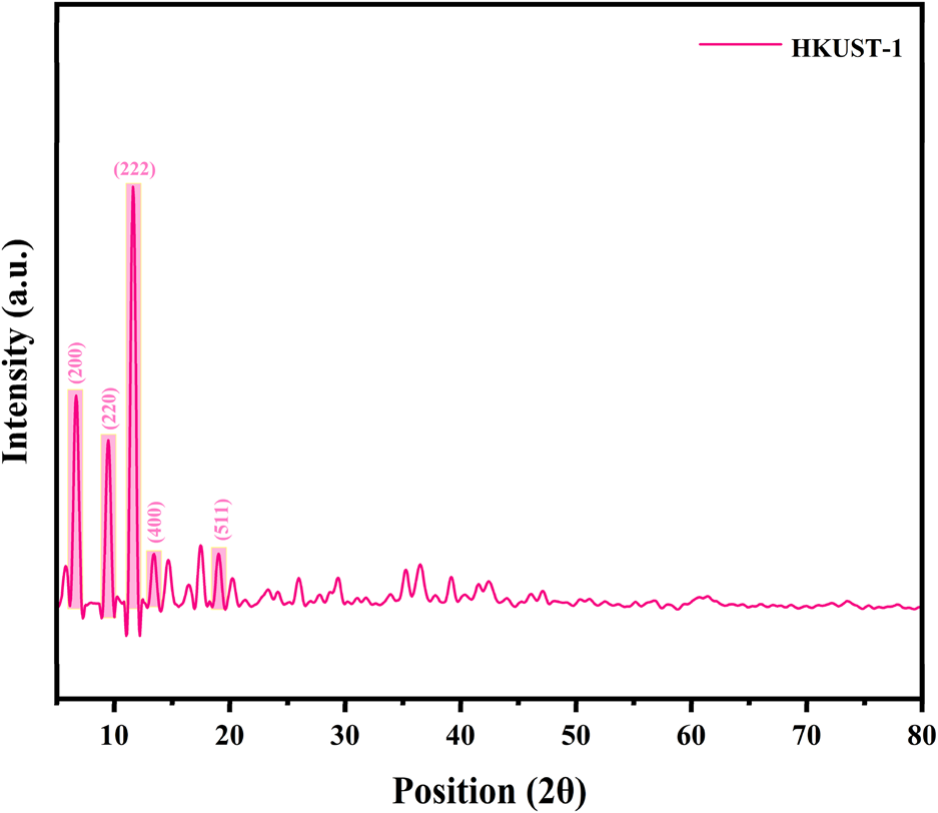
The XRD pattern of HKUST-1.

**Figure 4:**
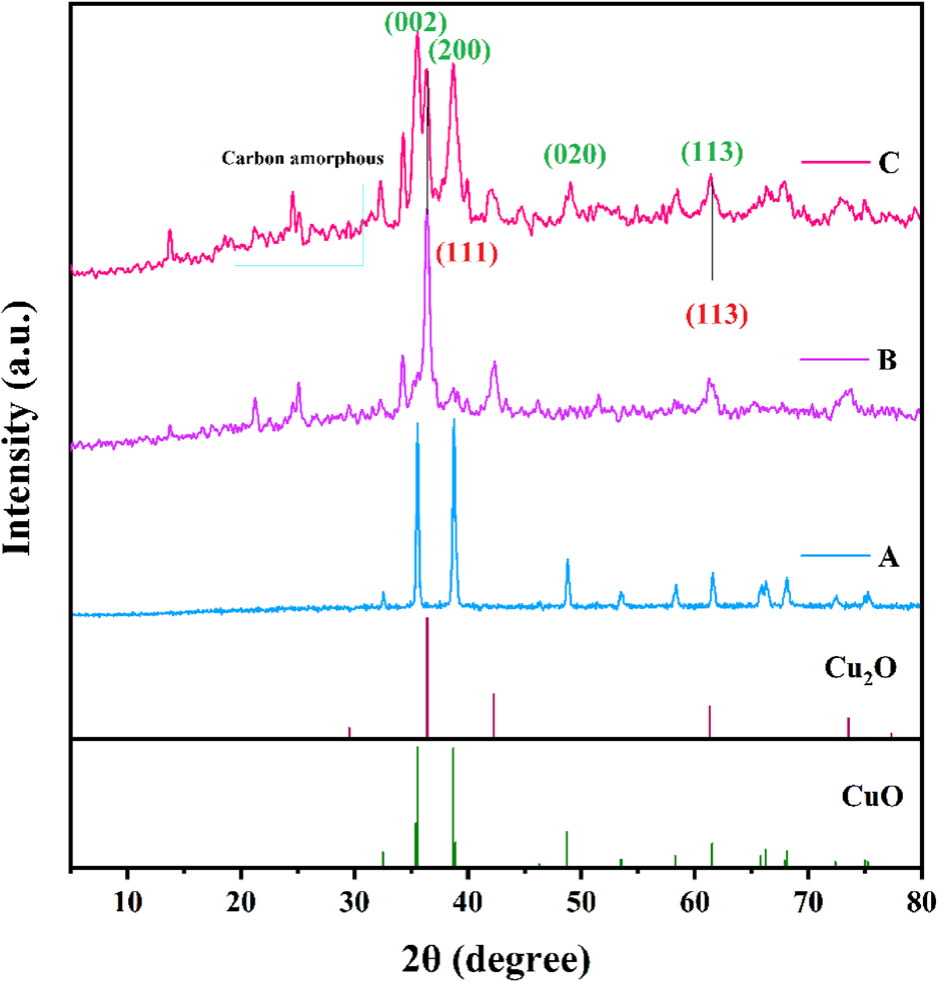
The XRD patterns of Cu_x_O@CS-400 with ratio (H:D 1:5) (**A**), Cu_x_O@CS-400 with ratio (H:D 1:3) (**B**), and Cu_x_O@CS-400 with ratio (H:D 1:2) (**C**).

The crystallinity, phase behavior, and diffraction peaks of HKUST-1 and Cu_x_O@CS-400 materials at different ratios (H: D 1:2, 1:3, and 1:5) were recognized by XRD analysis, as depicted in Fig. 3 and 4. The XRD diffraction peaks of HKUST completely matched with literature reports^47^. After pyrolysis the crystalline nature of the HKUST-1 has changed and new peaks have been created as follow: the as-prepared catalysts exhibited a broad peak at 2θ = 20–30˚, which is attributed to the carbon amorphous part of the as-synthesized samples (Fig. 4A–C is the XRD image of Cu_x_O@CS-400 material). Matching to the JCPDS standard card, the peaks at )002) (200) (220), and (113) crystal planes are the diffraction peaks of CuO (JCPDS: 48–1548), and the peaks appearing at (111) and (113) crystal planes in Fig. 4 belong to the diffraction peaks of Cu_2_O (JCPDS: 05–0667), which were clearly and precisely indicated in the XRD schemes of the samples^49^. It is noteworthy that the Bragg diffraction peaks of all oxides have a low vehemence, informing that Cu_x_O indicates negligible crystallinity with a small granule scale of 34 nm.

For samples in Fig. 4A–C, the characterization results show a transient shift in the refraction summit circumstances and relative intensities. These are caused by varying copper oxide loading ratios in the final materials as a result of varying HKUST-1 and diphenyl disulfide ratios used as precursor materials for sulfur doping in catalyst structures.

The sample with a ratio of 1:2 (H:D) was chosen as the required catalyst, and the subsequent analyses were carried out on it since the catalytic activity of the three manufactured samples (with varying ratios of diphenyl disulfide) had the same catalytic activity.

#### EDX analysis

**Figure 5:**
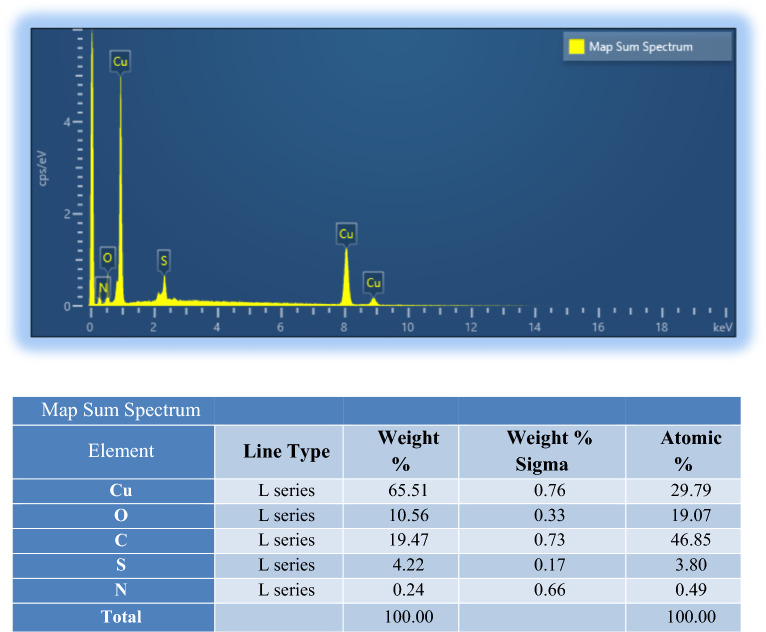
EDX data of Cu_x_O@CS-400.

The elemental composition of Cu_x_O@CS-400 was studied using EDX analysis, and the results manifested that copper, carbon, oxygen, and sulfur are presented in the particular selected area of the catalyst. This is a verification of the prosperous synthesis of the desired catalyst with high purity (Fig. 5). Moreover, the exact loading of copper in Cu_x_O@CS-400 using the AAS (atomic absorption spectroscopy) technique was found to be 65.5%, which increased upon pyrolysis of pre-material in the N_2_ atmosphere in comparison with HKUST-1. In addition, the amount of copper is in agreement with the EDX analysis.

**Figure 6:**
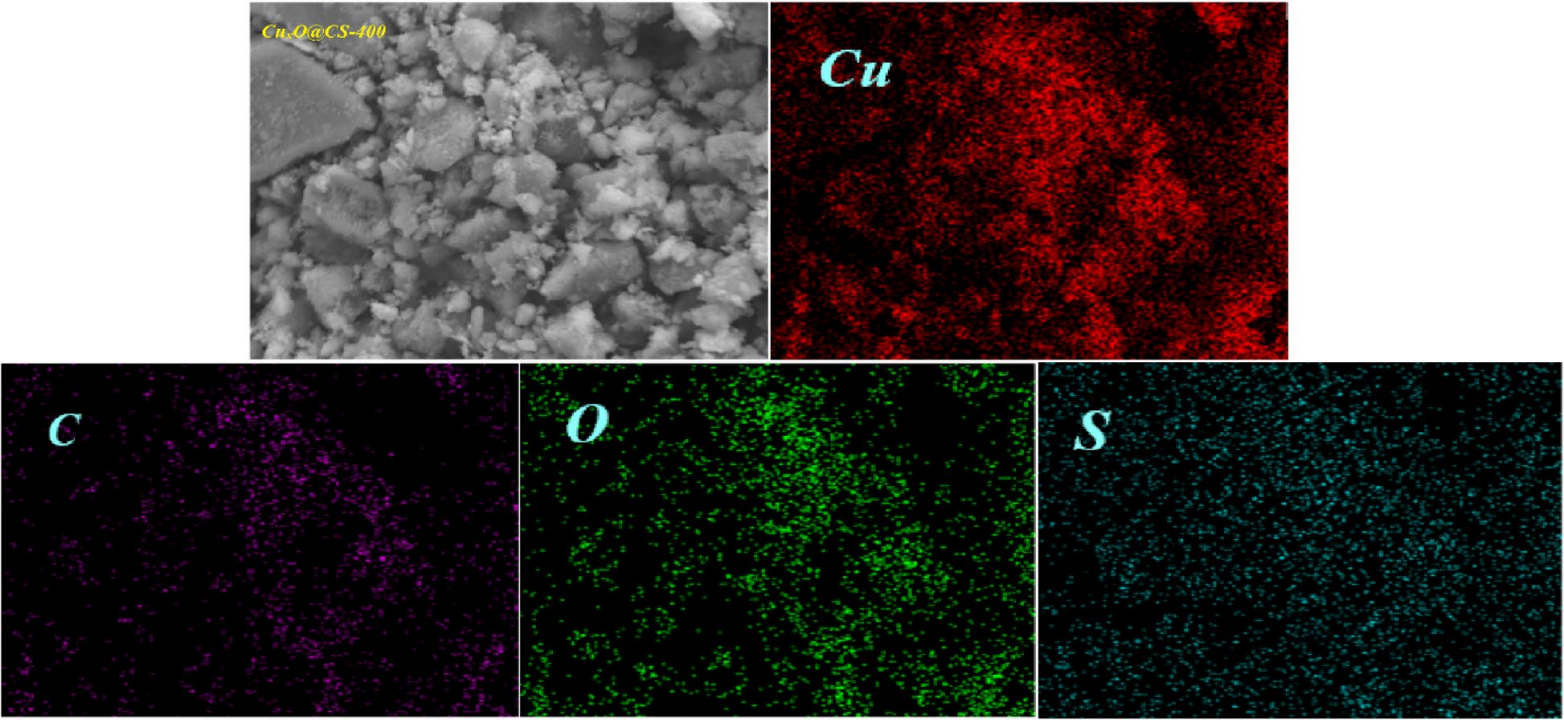
Elemental mapping images of Cu_x_O@CS-400 for Cu, C, N, O, and S elements.

Elemental mapping images of Cu_x_O@CS-400 are shown in Fig. 6, which obviously shows a homogeneous distribution of the desired elements Cu, C, O, especially S, in the structure of the catalyst.

#### Thermogravimetric analysis

**Figure 7:**
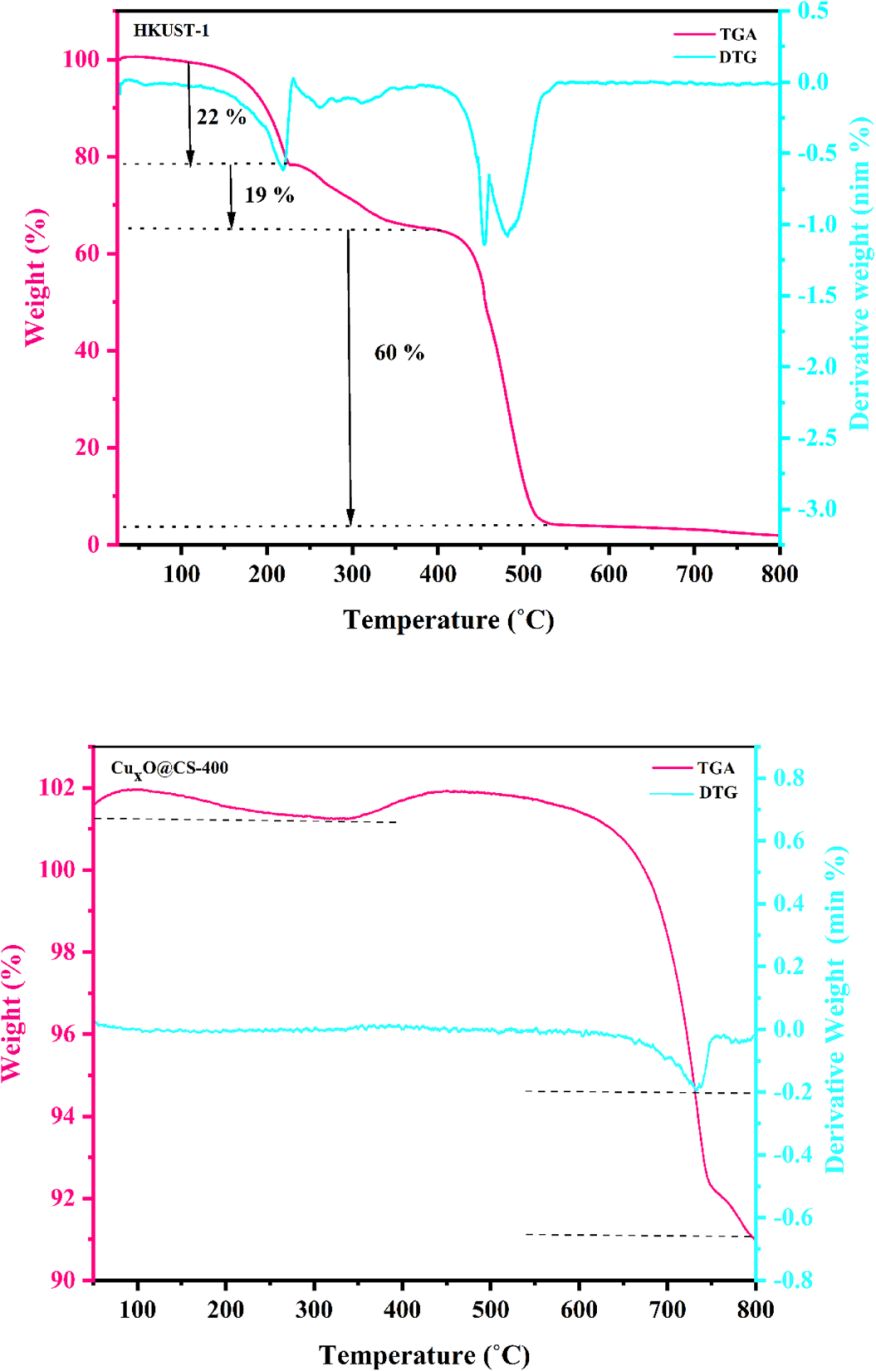
The thermogravimetric curves of HKUST-1 and Cu_x_O@CS-400 catalyst.

Thermal gravimetric analysis data at HKUST-1 and Cu_x_O@CS-400 were measured under the air with the heating speed of 10 °C min^−1^, and outcomes are shown in Fig. 7. The HKUST-1 exhibited three weight loss stages. Two primary degradations, nearly 22 and 19%, befell at 30–400˚C and were ascribed to the evaporation of organic solvents inside the pores, either adsorbed or coordinated water. The second degradation was observed from 400 to 550 °C, nearly 60%, owing to the destruction of the HKUST-1 framework^47^. The TGA and DGA analysis of prepared Cu_x_O@CS-400 concluded that the catalyst exhibited three weight loss stages. Two primary degradations within 50–450 °C with weight-loss about 4% and 6.75% was due to the loss of adsorbed water as well as the coordinated water molecules in the carbon substrate. The third destruction was coupled with a weight-loss about 10.23% between 450 and 750 °C suggesting that residue organic substances has started to decompose and produce some volatiles materials at 450 °C.

The TGA and DTG analyses of prepared Cu_x_O@CS-400 concluded that the catalyst is stable up to 750 °C without any significant weight loss (Fig. 7), which lays a solid foundation for convenient catalytic high temperature synthesis reactions.

#### FE-SEM and TEM analysis

**Figure 8:**
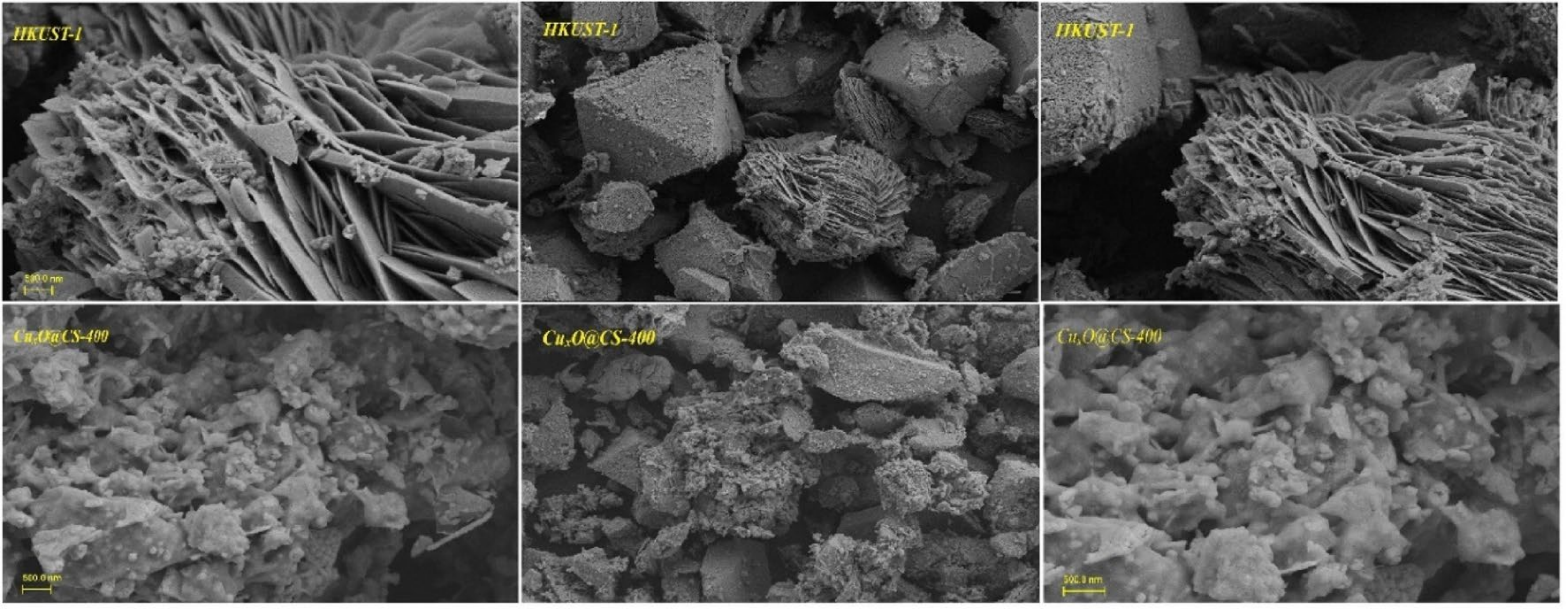
The FE-SEM images of HKUST-1 and Cu_x_O@CS-400 catalyst in different magnifications.

Figure 8 exhibits the scanning electron microscopy (FE-SEM) images of HKUST-1 and Cu_x_O@CS-400. The FESEM image of HKUST-1 shows a octahedral shaped morphology, as previously reported in the literature^47^. Upon incorporation of diphenyl disulfide and annealing of the pre-material at 400 °C, the structure of HKUST-1 is partially maintained and presents a rough surface due to the decoration of a number of Cu_x_O nanoparticles and holes that are produced during the carbonization process.

**Figure 9:**
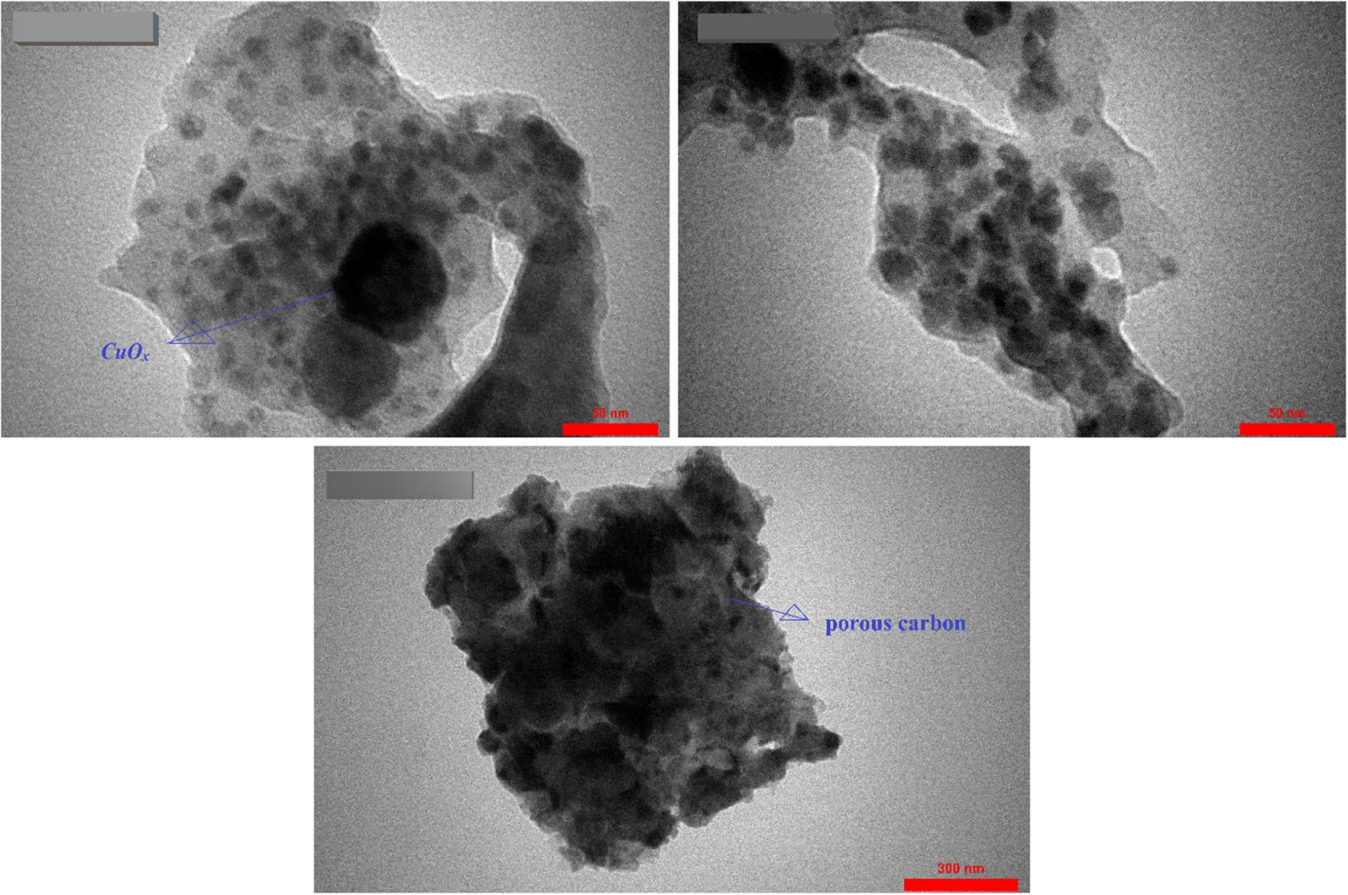
TEM images of Cu_x_O@CS-400 catalyst.

The structural examination was then assessed in depth using TEM measurements. The scattered Cu_x_O nanoparticles enclosed in the carbonaceous matrix are plainly visible in the concentrated black region. The porous carbon matrix that encircles the framework of dense metal oxides is indicated by the lighter portion of the catalyst (Fig. 9).

#### N_2_ adsorption–desorption isotherm

**Figure 10:**
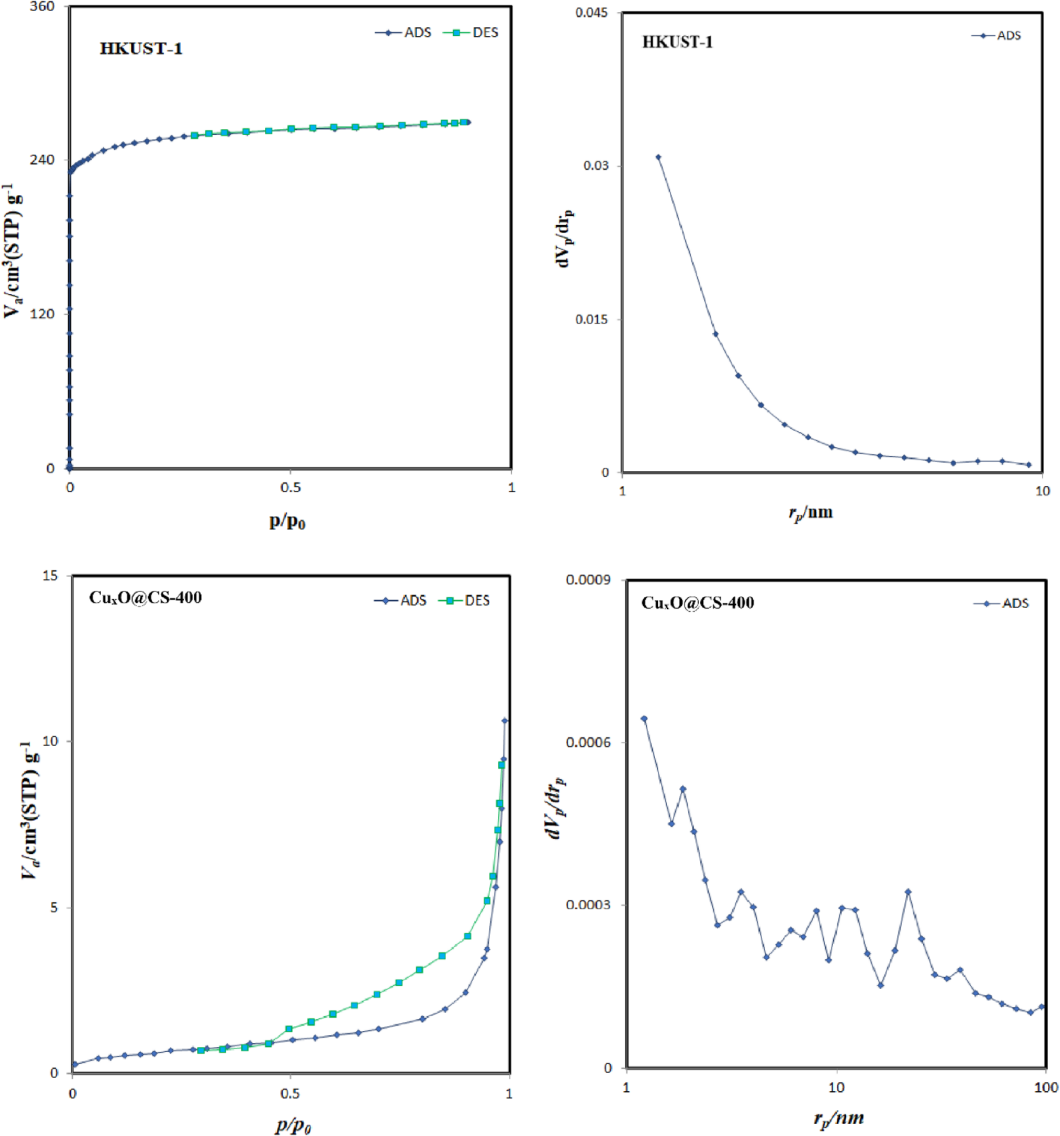
The N_2_ adsorption–desorption isotherms of HKUST-1 and Cu_x_O@CS-400 (H:D 1:2) catalyst.

According to IUPAC, the N_2_ adsorption–desorption isotherms of HKUST-1 and catalyst belonged to type IV isotherms, with the H_1_ hysteresis loop within the relative pressure of 0.1–0.95 as presented in Fig. 10. The specific surface areas of HKUST-1 and as-synthesized catalyst were 1021.2 and 2.4221 m^2^/g, respectively. It may be due to the agglomeration of Cu_x_O nanoparticles during the pyrolysis. The pore size was mainly distributed at 0.4173 and 0.01067 m^3^/g indicating a mesoporous structure. Finally, the average pore size of the catalysts (with different ratios of diphenyl disulfide) was calculated as 1.6345 and 26.826 nm, respectively.

### Catalytic performance of Cu_x_O@CS-400 in the nitroarenes hydrogenation

To better understand the catalytic efficacy of Cu_x_O@CS-400 nano-catalyst, the 4-nitro aniline has been selected as a catalytic model and subjected to reduction of the nitro aromatic compound in the presence of NaBH_4_. In order to check the progress of the reaction after the supplementation of the reaction, soft stratum chromatography (TLC) was performed. Different reaction parameters, including the solvent, the reaction temperature, dosage of the catalyst, and the reductant, were optimized. The reaction consequences are exhibited in Table 1.

Among the examined solvents (entries 8, 9, 10, and 11), the best conversion is obtained in the presence of H_2_O (entry 6, 100% conversion). According to a literature review, the presence of water molecules has a significant impact on the conversion of 4-nitro aniline to 1,4-diaminobenzene^50^. The reducing agent screening and its amount show that NaBH_4_ is present for the optimal conversion (entry 6). Higher or lower values of the reducing agent result in a reduction in the reaction yield (entries 12–14). Additionally, the impact of reaction temperature was examined (entries 12). The ideal temperature was determined to be 55 °C for the process. The ideal amount of catalyst for the reduction of 4-nitro aniline in the studied process was found to be 7 mg, or 0.072 mol% Cu, based on catalyst screening (entry 6). It should be noted that the reaction stops and no yield is produced in the absence of a catalyst (entry 1). Additionally, the decreased catalytic activity of CuxO@C-400 and HKUST-1 in the absence of the heteroatom demonstrated the critical role that sulphur and copper oxides played in the reaction's progression and final product yield (entries 3 and 4). Furthermore, while employing the sulfur-doped carbon substrate as the catalyst, no product was seen (entry 2).

The catalytic activity of Cu_x_O@CS-400 is examined in relation to other reactants. Different types of substituents on nitro aromatic compounds were transferred to aromatic amines under optimal reaction conditions.

In order to investigate the generality of the transformation, different types of aromatic nitro compounds, derivatives containing electron-donating (OMe, OH, NH_2_) and electron-withdrawing (Br, CF_3_, Cl, CN) were reduced under the optimized reaction terms. The supreme returns of the obtained aromatic amines in a very short reaction time indicate the good efficiency of the catalyst in both electron-poor and electron rich substituted nitro aromatic compounds (Table 2). Unfortunately, the yields of the corresponding reduced products were low for aliphatic nitro compounds.

**Table 2:** Reduction of 4-nitro aniline in different conditions^a^.


Entry	Catalyst (mg)	Solvent	Temp °C	Reducing agent (equiv.)	Time (min)	Yield (%)^b^
1	None	H_2_O	55	NaBH_4_ (2)	25	–
2	CS-400	H_2_O	55	NaBH_4_ (2)	25	–
3	HKUST-1	H_2_O	55	NaBH_4_ (2)	25	49
4	Cu_x_O@C-400	H_2_O	55	NaBH_4_(2)	45	55
5	Cu_x_O@CS-400 (3.5)	H_2_O	55	NaBH_4_ (2)	28	100
6	Cu_x_O@CS-400 **(7)**	**H**_**2**_**O**	**55**	**NaBH**_**4**_** (2)**	**20**	**100**
7	Cu_x_O@CS-400 (14)	H_2_O	55	NaBH_4_ (2)	13	100
8	Cu_x_O@CS-400 (7)	H_2_O/EtOH	55	NaBH_4_ (2)	5	100
9	Cu_x_O@CS-400 (7)	MeOH	55	NaBH_4_ (2)	20	95
10	Cu_x_O@CS-400 (7)	Toluene	55	NaBH_4_ (2)	20	trace
11	Cu_x_O@CS-400 (7)	THF		NaBH_4_ (2)	20	54
12	Cu_x_O@CS-400 (7)	H_2_O	75	NaBH_4_ (2)	10	100
13	Cu_x_O@CS-400 (7)	H_2_O	55	NaBH_4_ (1)	31	100
14	Cu_x_O@CS-400 (7)	H_2_O	55	NaBH_4_ (3)	15	100
15	Cu_x_O@CS-400 (7)	H_2_O	55	N_2_ H_4_. H_2_O (2)	70	98
16	Cu_x_O@CS-400 (7)	H_2_O	55	N_2_ H_4_. H_2_O (3)	62	98

^a^Reaction condition: 4-nitro aniline (1 mmol), NaBH_4_, Solvent (3 mL), catalyst.

^b^Isolated yield.

E.g. Significant values are in [bold].

For a more detailed investigation of the catalytic activity of Cu_x_O@CS-400 nano-catalyst, the investigated reaction was performed in the presence of 10 mmol of 4-nitro aniline under optimal reaction conditions, and the yield of the product was reported to be 95%.

### Mechanistic sight for the hydrogenation of the nitroarenes by CuxO@CS-400

**Figure 11:**
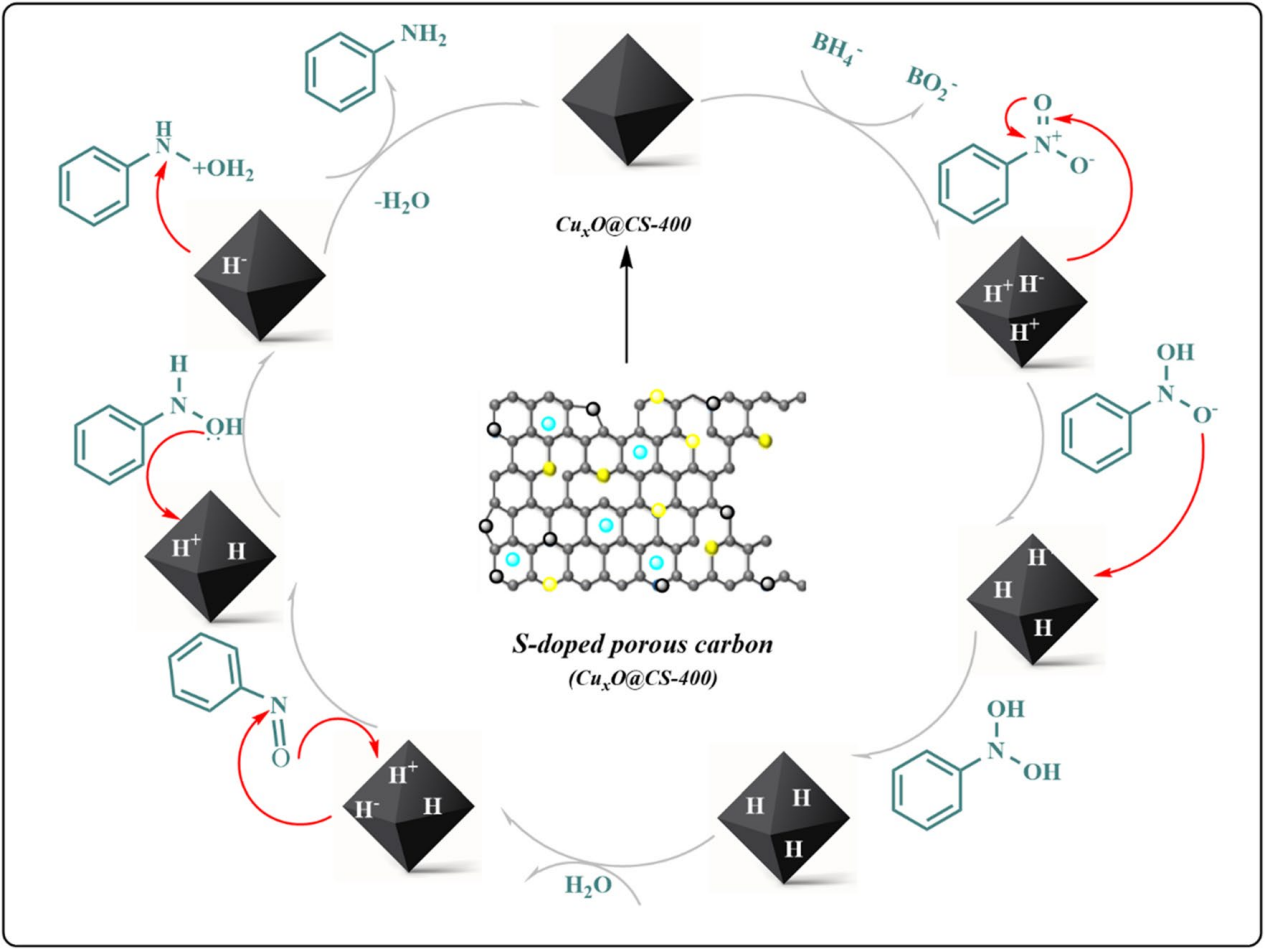
Mechanistic sight for the hydrogenation of the nitroarenes by Cu_x_O@CS-400.

According to the previous papers^43^, the plausible mechanistic pathway of the hydrogenation process of nitrobenzene was presented (Fig. 11). Firstly, the reaction between the reducing agent (NaBH_4_) and the solvent (H_2_O) can generate hydrogen (H_2_), which moved to the surface of the catalyst and the catalyst surface activation was done while NaBO_2_ was formed as the by-product at room temperature. In the next step, the adsorption of the substrate onto the activated CuxO@CS-400 surface was done to transfer the hydride from the catalyst to the nitrobenzene. Then the nitro reduction was happened and the nitroso group was produced. Subsequently, the hydroxylamine was generated according to the addition reductive process on nitroso group. In final step, the primary amine was released from the surface of the catalyst by hydrogenation of the hydroxylamine and the also the CuxO@CS-400 surface was set-free and for the next hydrogenation catalytic runs.

### Performance comparison of Cu_x_O@CS-400 in hydrogenation of aromatic nitro compounds with other reported MOF-derived Cu-based catalysts

In this part of the research work, the catalytic efficiency and performance of Cu_x_O@CS-400 in the hydrogenation reaction of nitroarenes were reconciled with the previously published MOF-derived Cu-based catalysts, and the outcomes are succinct in Table 3. However, every one of these catalysts shown in Table 3 has its own advantages. It is clear that the above catalyst has shown significant activity and efficiency due to reaction conditions such as short reaction time, green solvent (H_2_O), low temperature, appropriate amount of reductant, recovery and reusability, as well as time and product yield.

**Table 3:** Checking the catalytic power of Cu_x_O@CS-400 with some MOF-derived Cu-based catalysts in the reduction reaction of para-nitro aniline.

Entry	Catalyst	Reaction conditions	Time (h)	Yield (%)	Refs.
1	Cu_x_O@CS-400 7 mg	4-Nitro aniline (1 mmol), NaBH_4_ (2 mmol), H_2_O (3 mL), 55 °C	20 min	100	This article
2	Cu@C-400 15 mg	Nitrobenzene (2 mmol), EtOH (10 mL), NaBH_4_ (4 mmol, added in two times), 45 °C	30 min	100	^50^
3	Cu/Cu_2_O/C 0.04 mg	4-Nitrophenol (5 × 10^–3^ M), NaBH_4_ (0.2 M), H_2_O (2 mL), RT	2.67 min	99	^51^
4	Cu/Cu_2_O@C 10 mg	4-Nitroaniline, NaBH_4_ (0.1M), H_2_O, RT	1	100	^52^
5	Cu/Cu2O@C-rGO 1 mg	4-Nitrophenol (0.3 × 10^–3^ mmol), NaBH_4_ (0.25 mmol), H_2_O (2 mL), RT	90	98	^53^
6	a-Cu@C 5 mol %	Nitrobenzene (1 mmol), THF/H_2_O (1:2. 3 mL), NaBH_4_ (3 mmol), 50 °C	4	98	^54^

### Recyclability and stability of Cu_x_O@CS-400

**Figure 12:**
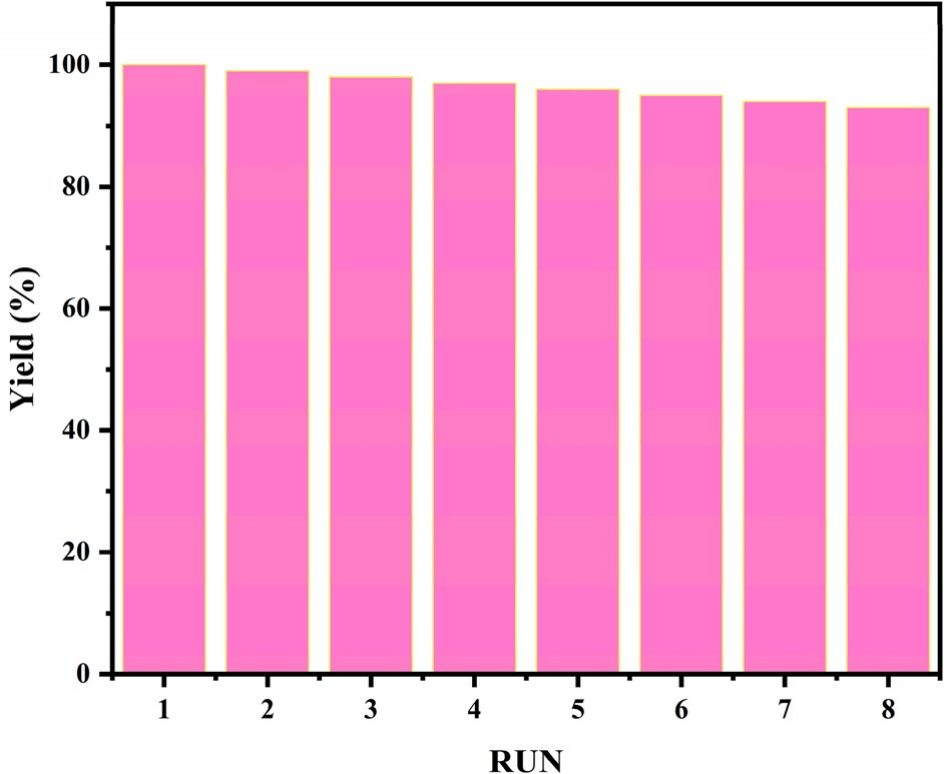
The recycling of the CuO_x_@CS-400 catalyst.

Considering the environmental and economic advantages and the reusable characteristics of a catalyst, it is very important for economic and industrial applications. Accordingly, the reusability of Cu_x_O@CS-400, under optimal reaction conditions was examined for the transformation of 4-nitroaniline to 1,4-phenylenediamine. The reaction vessel was supplemented with a fresh sample of 4-nitroaniline and the repurposed catalyst. When the reaction mix was stirred under ideal circumstances, 90% yield was produced after eight cycles, suggesting that Cu_x_O@CS-400 could be recycled for the reduction of 4-nitro aniline (Fig. 12). After the eight cycles, the product formation efficiency decreased to 86%, which could be due to copper oxide leaching. So, atomic absorption spectroscopy was performed on recycled catalyst and the percentage of copper was determined to be 64.1%, which indicates 1.4% decrease compared to the fresh catalyst.

**Figure 13:**
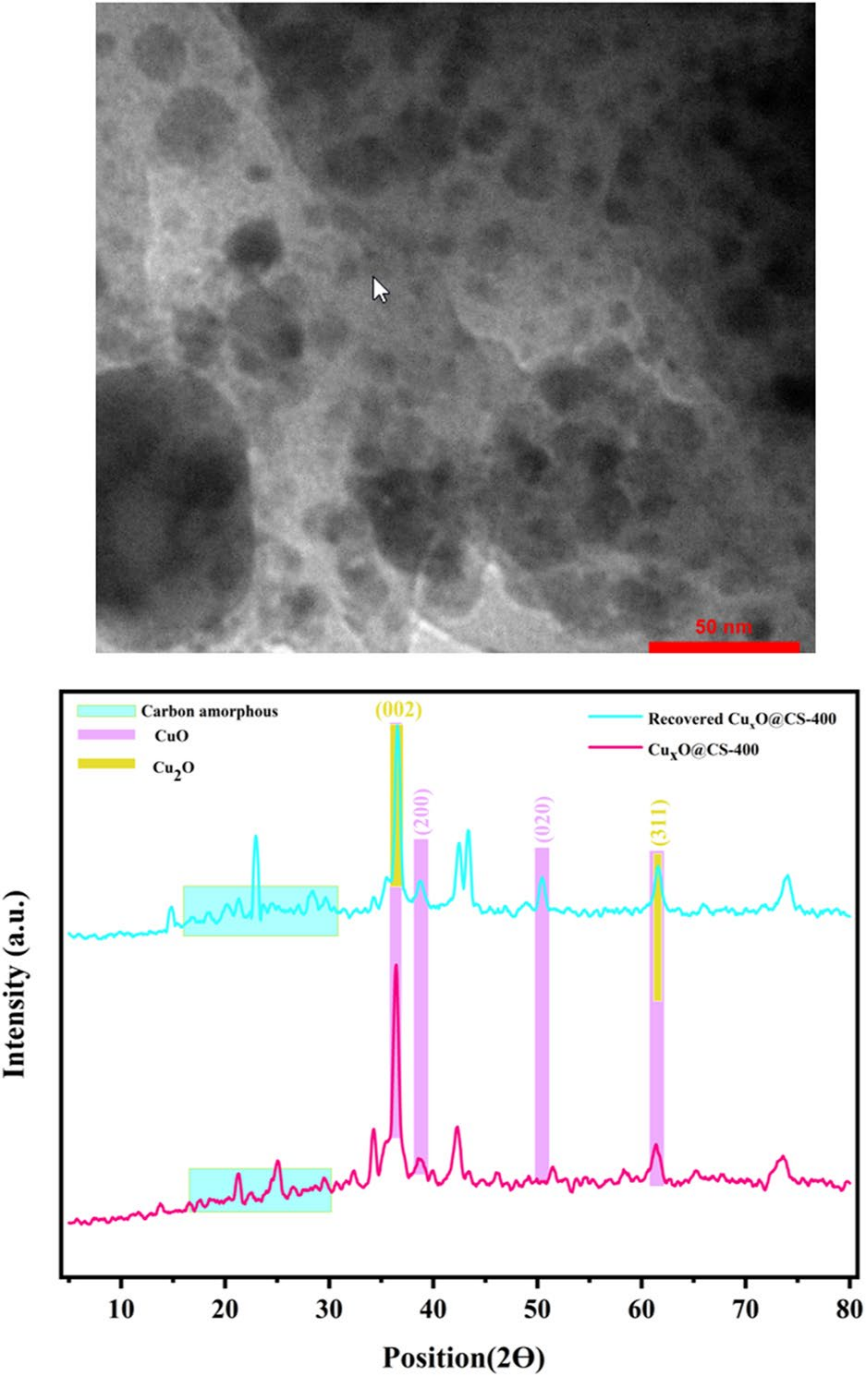
TEM image and XRD patterns of fresh and recovered Cu_x_O@CS-400 after the 8th cycle.

To prove the stability and high activity of the Cu_x_O@CS-400 catalyst, several stages of recovery were done, and finally, the structural characteristics and morphology of the recovered catalyst were compared with those of the fresh catalyst by accomplishing XRD and TEM analysis following the reusability examination (Fig. 13). This analysis indicated that the morphology of the Cu_x_O@CS-400 catalyst used was relatively similar to the catalyst without recovery, which proves and confirms that it is a powerful catalyst (Fig. 13).

## Conclusion

In conclusion, a sulfur doped HKUST-1 material was subjected to pyrolysis at 400 °C in a nitrogen atmosphere, resulting in the creation of an exquisite heterogeneous nano-catalyst. This novel heterogeneous catalyst, named Cu_x_O@CS-400, underwent comprehensive characterization and exhibited outstanding performance in the reduction reaction of nitro aromatic compounds. It also demonstrated excellent recovery capability, with a yield of 90% over eight consecutive cycles. Furthermore, even after the eighth cycle, Cu_x_O@CS-400 keeps its original morphology, indicating its long-lasting nature. The catalyst offers several advantages, including its simple synthesis, short reaction time, eco-fraternal disposition, thermal resistance, and remarkable catalytic activity and reusability. These attributes make Cu_x_O@CS-400 a highly desirable catalyst for various catalytic applications.

## Data Availability

All data generated or analyzed during this study are included in this published article.
